# Influence of the scleral indentation technique on the re-detachment rate following retinal detachment surgery

**DOI:** 10.1186/s40942-022-00362-8

**Published:** 2022-02-15

**Authors:** Philip Wakili, Karl T. Boden, Peter Szurman, Annekatrin Rickmann, Rosemarie Schlosser, Lukas Bisorca-Gassendorf, Kai Januschowski

**Affiliations:** 1 Eye Clinic Sulzbach, Knappschaft Hospital Saar, An der Klinik 10, 66280 Sulzbach, Germany; 2 Centre for Ophthalmology, University Eye Hospital Tuebingen, Schleichstraße 12, 72076 Tuebingen, Germany

**Keywords:** Vitreoretinal surgery, Scleral indentation, Retinal detachment, Pars plana vitrectomy, Surgical techniques

## Abstract

**Purpose:**

The aim of this study was to determine whether the choice of scleral indentation technique during primary rhegmatogenous retinal detachment surgery has an influence on the risk of re-detachment.

**Methods:**

We included retrospectively 154 eyes with a primary rhegmatogenous retinal detachment treated in the Eye Clinic Sulzbach/Saar Germany, who were operated on by two experienced surgeons using the same basic surgical setup. Surgeon A performed an external 360° indentation, shaved the vitreous base using the light pipe cap, and used the operating microscope (opm) for direct visualization. Surgeon B performed an external 360° indentation, shaved the vitreous base using a simple indentor, and used an endoillumination (light pipe) with the opm and a handheld widefield lens for direct visualization.

**Results:**

Comparing both indentation procedures, 15.66% (13/83) of patients operated on by surgeon A and 9.86% (7/71) of patients operated on by surgeon B had a retinal re-detachment within a follow-up period of 6 months (adj. *p* = 0.64, two-proportion Z-test).

**Conclusion:**

The rate of retinal re-detachment could be influenced by the indentation technique at the end of surgery favoring external indentation and internal visualization with an endoilluminator (chandelier light). We attribute this to the better visualization of the vitreous base facilitated by endoillumination. However, many variables play a role in the development of retinal re-detachment, requiring further studies with a larger number of patients.

**Supplementary Information:**

The online version contains supplementary material available at 10.1186/s40942-022-00362-8.

## Key messages

What we already know:

- Scleral indentation is widely recognized as an important step in rhegmatogenous retinal detachment surgery via pars-plana vitrectomy.
- There are several surgical techniques for scleral indentation, however the procedures are neither standardized, nor have they ever been compared to one another to the best of our knowledge.
  What this study adds:
- This study shows that the rate of retinal re-detachment could be influenced by the indentation technique at the end of surgery favoring external indentation and internal visualization with an endoilluminator (chandelier light).
- We attribute this to the better visualization of the vitreous base facilitated by endoillumination.

**Supplementary Information:**

The online version contains supplementary material available at 10.1186/s40942-022-00362-8.

## Introduction

The surgical treatment of retinal detachment has improved significantly as a result of the introduction and technical refinement of pars plana vitrectomy [1–4]. However, re-detachments are still a challenge. Although many attempts to improve the re-detachment rate have been performed, it still varies between 10 and 30% [5–7]. Perioperative risk factors that could improve this unfavorable outcome are being investigated at the moment; however, no consensus about the best surgical approach has been reached [8, 9]. The majority of surgeons are using vitrectomy for most cases [10], while they perform scleral buckling for selected cases only. Some surgeons advocate for a combination of both methods [11] and others prefer cryocoagulation over lasercoagulation, but the majority prefer lasercoagulation [6, 7]. There is also a vivid discussion surrounding the use of endotamponades (gases and silicone oils) and heavy liquids. In brief, there are many ways to surgically address a retinal detachment; however, there is no consensus on the most successful approach [12]. Our aim was to address one particular aspect of the surgery: scleral indentation. Scleral indentation is usually performed while shaving the vitreous base [13]. Traditionally, meticulously vitreous shaving is considered an important step in retinal detachment surgery, since remnants of the vitreous base are associated with an induction of proliferative vitreoretinopathy (PVR) [6, 7, 14, 15]. There are several surgical and visualization techniques that can be used to remove as much of the vitreous base as possible: one can either use an external indentation with a regular light-pipe endoillumination or a chandelier, combine a chandelier with an external illuminating source (light pipe with cap), or use the standard operating microscope (opm) without a lens [16].

Considering the aforementioned variables, it is questionable whether a consensus about a standardized surgical approach can be reached with enough solid scientific evidence supplied by a randomized controlled trial (RCT), for example. Therefore, we decided to compare only two methods of indentation in a retrospective, yet semi-standardized setting: classic external indentation with a regular light-pipe endoillumination using a light pipe and contact lens visualization versus external indentation with a capped light pipe under direct visualization via the opm, without using a contact lens.

## Materials and methods

### Subjects

This retrospective study was approved by the Ethics Committee of the Saarland Medical Association (approval number 243/14) and was in accordance with the 1964 Helsinki declaration and its later amendments. Written informed consent was obtained from all patients.

We included all patients (154 eyes from 154 patients) with a primary rhegmatogenous retinal detachment treated in the Eye Clinic Suzbach/Saar Germany between 2015 and 2017 who were operated on by two experienced surgeons using the same basic surgical setup. All patients received a pars plana vitrectomy (PPV) without the use of buckling surgery. Preoperative evaluation included best-corrected visual acuity (BCVA), intraocular pressure (IOP), a full slit-lamp and fundus examination, a fundus drawing, and a spectral domain optical coherence tomography scan (SD-OCT) (Heidelberg Engineering, Germany). Only patients with a primary rhegmatogenous retinal detachment were included into this study. The symptom duration in our patient collective did not exceed two weeks. Patients with previous retinal re-detachment and tractional retinal detachment, caused by advanced diabetic eye disease, for example, were excluded. We also excluded patients with high myopia (spherical equivalent below -6 diopters or axial length above 26.5 mm), posttraumatic eyes and strong PVR reactions (exceeding PVR grade B in the Retina Society classification).

A standard procedure in surgical emergencies (per se) is rather difficult, and the surgical evolution during each surgery can differ. The “surgeon factor” is a possible influence; consequently, we chose to engage two specialized retinal surgeons who had each performed well over 2000 vitrectomies. Both surgeons used the same vitrectomy system and the same opm with similar settings. For the purpose of improved standardization of our analysis, we decided to only include primary retinal detachments that were considered uncomplicated by the operating surgeon.

To ensure the safety and efficacy of the postoperative position after rhegmatogenous retinal detachment, the postoperative position was directly communicated to the patient by the surgeon and periodically monitored by the medical staff.

### Pars plana vitrectomy (PPV)

**Fig. 1:**
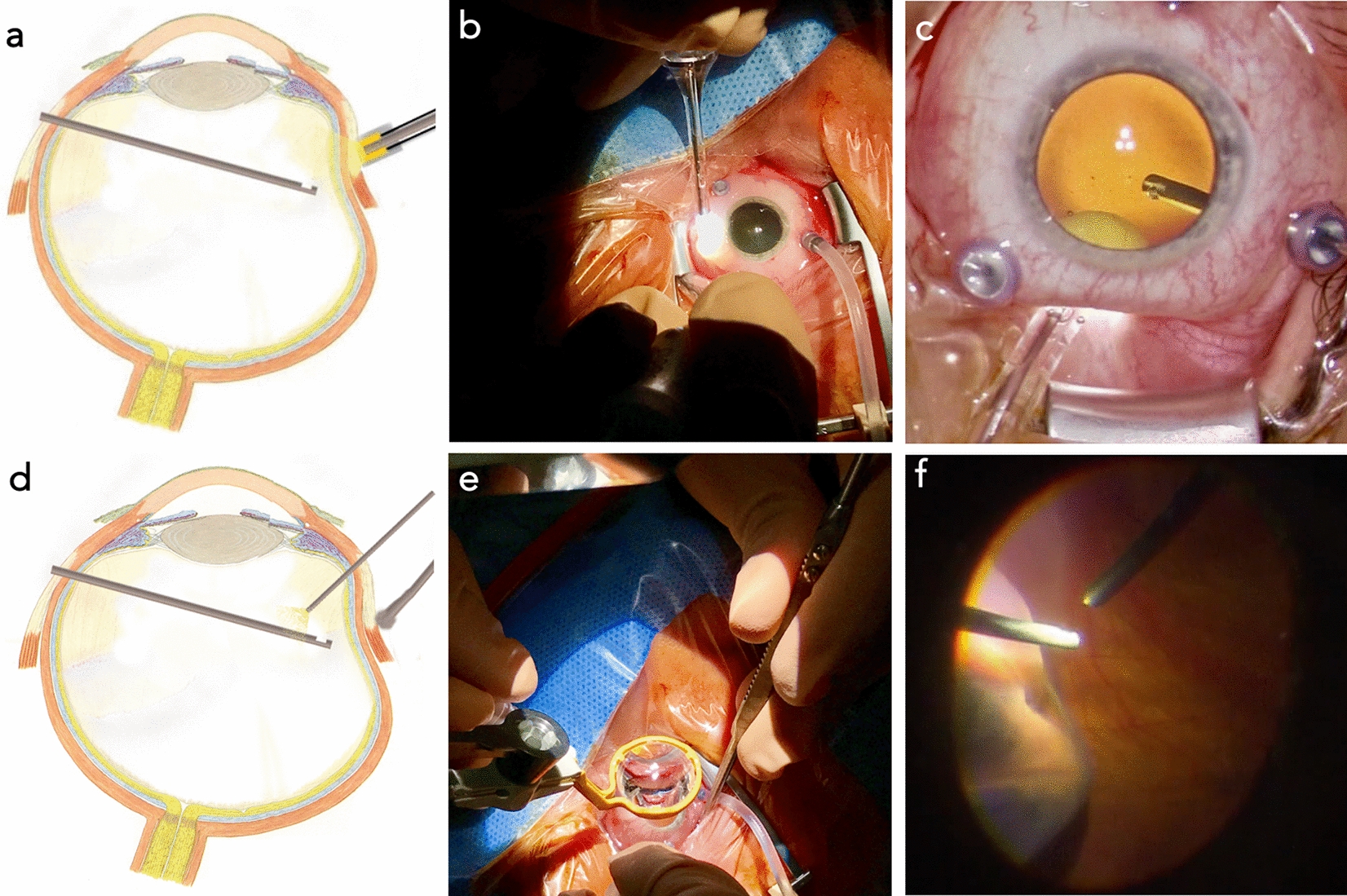
Comparison of both methods for scleral indentation: Sections **a**–**c** represent the method used by surgeon A. Sections **d**–**f** represent the method used by surgeon B. **a**, **d** show schematic cross-sectional representations of the eyeball during each indentation method. In both cases the vitrectome is held intravitreally. In **a**, the light pipe cap serves as for indentation, while in **d**, a simple indentor is used with a chandelier light being held intravitreally for better visualization. **b**, **e** show overview images of the surgical fields of both indentation techniques, while **c**, **f** show a more detailed view from the perspective of the respective surgeon

PPV was performed under a standard ophthalmic operating microscope (Lumera 700 microscope, Carl Zeiss Meditec AG, Germany) by two selected, experienced surgeons. A standard 23-gauge sutureless vitrectomy system was used (EVA, D.O.R.C., Netherlands). The endoillumination was set to 80%, and a two-dimensional cutter (TDC Cutter 23G, D.O.R.C., Netherlands) was set to 8000 cpm during core vitrectomy (maximum vacuum 450 mmHg), peripheral vitrectomy (maximum vacuum 250 mmHg), and shaving of the vitreous base for all procedures. All patients had a posterior vitreous detachment. A core vitrectomy was performed, followed by a reattachment of the retina using ultrapure perfluorocarbons (F-Octane 1.76 g/cm^3^ C_8_F_18_, Geuder GmbH, Germany) under visual control. Thereafter, Surgeon A performed an external 360° indentation and shaving of the vitreous base using the light cap as indentation and direct visualization with the opm with a less aggressive peripheral shaving setting (maximum vacuum 300 mmHg, flow 15 cc/min, and cutter rate of 8000). Surgeon B performed an external 360° indentation and shaving of the vitreous base using a simple indentor under direct visualization with an endoillumination (light pipe) using an opm and handheld lens (OLV2 Ocular Landers HRI Vitrectomy Lens, Ocular Instruments, USA) and the same settings. Figure 1 shows a comparison of both methods for scleral indentation. At the end of surgery, endotamponade with hexafluoroethane (C_2_F_6_), sulfur hexafluoride (SF_6_), or air was used. Finally, the infusion cannula was removed, and the sclerotomies were sutured if necessary (Vicryl 7–0, Johnson & Johnson Intl., USA). The patients were instructed to adhere to a position that would allow the ideal effect of the tamponade vector.

### Statistical analysis

Statistical analyses were conducted with R 3.4.3. Proportions were compared using a two-proportion Z-test with continuity correction to assess the significance of the differences (prop.test function). Resulting *p*-values were adjusted for multiple testing by Benjamini–Hochberg correction (p.adjust function).

## Results

This retrospective study included 154 eyes of 154 patients who underwent operation for a primary retinal detachment between 2015 and 2017. The mean age was 64.81 (± 10.74) years; 42 patients were female and 112 male. Eighty-two eyes were phakic, while 72 were pseudophakic. Patients were followed up over a period of 6 months.

All 154 eyes with a retinal detachment underwent PPV and received a gas or air tamponade at the end of surgery. In all operations, there was no case of iatrogenic lens touch.

Of those eyes, 111 were treated with C_2_F_6_ gas, 38 with 20% SF_6_ gas, and 5 with air. The re-detachment rate of patients receiving C_2_F_6_ gas was 10.39%, those receiving SF_6_ gas had a re-detachment rate of 2.60%, and the retina of all five eyes treated with an air tamponade did not re-detach.

Surgeon A operated on 83 eyes with external indentation using a capped light pipe under the opm without a contact lens, and Surgeon B operated on 71 eyes with external indentation with regular light pipe endoillumination and a widefield contact lens visualization.

A retinal re-detachment was observed in 15.66% of eyes (13 of 83) in the group operated on by Surgeon A and 9.86% of eyes (7 out of 71) in the group operated on by Surgeon B, within a follow-up period of six months (adj. *p* = 0.64, two-proportion Z-test).

Of the 83 eyes operated by Surgeon A with external indentation with a capped light pipe under the opm without using a contact lens, 75 were injected with C_2_F_6_, 5 with SF_6_, and 3 with air. In the C_2_F_6_ subgroup, 13 eyes had a retinal re-detachment, while in the other subgroups (SF_6_ and air tamponade) all eyes were successfully treated without the occurrence of a retinal re-detachment. Thirty-six eyes were injected by Surgeon B with C_2_F_6_, 33 with SF_6_, and 2 with air. A retinal re-detachment occurred in three eyes in the C_2_F_6_ subgroup, four in the SF_6_ subgroup, and none in the subgroup treated with air.

In the cases operated on by surgeon A, 56 (67.47%) eyes had retinal tears in the upper quadrants while in 12 (14.46%) eyes the retinal tears were located in the lower quadrants and in 15 (18.97%) cases the retinal tears were located in both hemispheres. The mean number of retinal breaks was 1.8.

In the cases operated on by surgeon B, 54 (76.05%) eyes had retinal tears in the upper quadrants while in eight (11.27%) eyes the retinal tears were located in the lower quadrants and in nine (12.69%) cases the retinal tears were located in both hemispheres. The mean number of retinal breaks was 2.11.

The mean value of the number of retinal tears was 1.77 in phakic patients and 2.04 in pseudophakia patients.

There were three patients operated on by surgeon A and seven patients operated on by surgeon B with a grade B PVR reaction (according to the Retina Society classification). We did not look at cases exceeding PVR grade B reactions.

Statistical correlation was performed and showed no significant differences between the two groups regarding macula status, combination with a phacoemulsification, rate of proliferative vitreoretinopathy grade C, extent of affected quadrants, and sex. Although Surgeon A had a significant tendency to use C_2_F_6,_ Surgeon B used SF_6_ more often (*p* < 0.05). Both surgeons used an air tamponade only in a few particular cases. Perioperative characteristics are shown in Table 1.

**Table 1:** Perioperative characteristics

Perioperative characteristics	Surgeon A	Surgeon B	Significance (adj. p)
Number of surgeries	83	71	
Male	63 (75.9%)	49 (69.01%)	0.44 (0.64)
Female	20 (24.1%)	22 (30.99%)	0.44 (0.64)
Rate of retinal re-detachment	13 (15.66%)	7 (9.86%)	0.41 (0.64)
Involvement of 1 quadrant	18 (21.69%)	17 (23.94%)	0.89 (1)
Involvement of 2 quadrants	45 (54.21%)	44 (61.97%)	0.42 (0.64)
Involvement of 3 quadrants	12 (14.46%)	9 (12.68%)	0.93 (1)
Involvement of 4 quadrants	8 (9.64%)	1 (1.41%)	0.07 (0.30)
Cases with detached macula	48 (57.83%)	38 (53.52%)	0.71 (0.92)
Combined cataract surgery	15 (18.07%)	7 (9.86%)	0.22 (0.57)
PVR	3 (3.6%)	7 (9.86%)	0.22 (0.57)
C_2_F_6_ gas tamponade	75 (90.36)	36 (50.70%)	1.24 × 10^–7^ (8.06 × 10^–6^)
SF_6_ gas tamponade	5 (6.03%)	33 (46.48%)	1.94 × 10^–8^ (2.52 × 10^–7^)
Air tamponade	3 (3.61%)	2 (2.82%)	1 (1)

## Discussion

With the modern surgical techniques performed for rhegmatogenous retinal detachment, especially PPV, very high success rates can be achieved by experienced surgeons. Therefore, even apparently trivial factors such as the method for scleral indentation might play an important role in the success rate and functional outcome after PPV. It is important to mention that the primary success of any retinal detachment surgery depends on identification of retinal breaks by a good clinical examination. In the cases presented here, the reason for retinal re-detachment was an existing retinal tear. However, it is not always easy to tell whether it is a new retinal tear or one that has been overlooked in the initial surgery.

In this study, we investigated for the first time two methods of scleral indentation with regard to the anatomical success rate in the treatment of a primary rhegmatogenous retinal detachment. Overall, the re-detachment rate is in the lower range with respect to the literature. Comparing the rate of re-detachments between the two surgical procedures, we showed that external indentation and internal visualization with an endoilluminator tends to be more favorable.

We attribute this to the better visualization of the vitreous base facilitated by the endoillumation. It can be assumed that the indentation by fiber optics with a special attachment does not lead to sufficient visualization of the vitreous base or retina. Consequently, the surgeon is rather predisposed to overlook vitreous tractions or retinal tears, which can lead to a re-detachment [17, 18]. Because of the light cap and scleral thickness, we created a larger distance for the light to reach its point of interest. Light is also absorbed while passing through the scleral tissue, further reducing illuminance [19, 20]. This issue might become less important with the introduction of new 3D visualization techniques that offer a digital improvement of illumination and contrast enhancement [21, 22].

Clear limitations of this study are the retrospective study design, the resulting short follow-up period and the surgeons’ different preferences for using endotamponades (Surgeon A used more C_2_F_6_, while Surgeon B used more SF_6_). In addition, aspects regarding the heterogeneity of the setting can be the source of biased results.

One might attribute the difference in endotamponade use to a possible underlying bias; C_2_F_6_ is mostly used in more complex cases of retinal detachment with a higher probability of re-detachments. Furthermore, a longer duration of endotamponade is associated with a higher success rate [23]. However, it is noteworthy that more than twice the number of patients operated on by Surgeon B developed PVR reactions with almost identical involvement of quadrants. Therefore, we attribute this fact more to the surgeon’s preference than to any other factor. It would certainly also have been interesting to have a subdivision into early and late re-detachment here, but in our study, retinal re-detachment occurred in all patients in the period of the first three months, why we did not create a subdivision.

Surgical skills are also thought to play an important role in the incidence of retinal re-detachments [17]. However, Surgeon A had a minor advantage over Surgeon B regarding surgical experience, hence we do not consider this fact to significantly alter our conclusion. It is also very unlikely that over the period of the retrospective analysis (approximately 2 years) a significant change in surgical experience or any type of technical change might have occurred. One advantage of this study was that the two surgeons involved came from identical surgical schooling backgrounds and operated almost identically except for the choice of indentation.

In addition, it should be considered that other baseline characteristics of retinal detachments, such as the complexity and chronicity (e.g. degree of PVR) of the retinal detachment, the presence of a high myopic refractive error, lens status, patient age, and the type of tamponade used, may have an impact on the re-detachment rate after the first PPV and overall on the success of the surgery.

We would conclude that not a particular type of illumination technique is solely responsible for the success of the surgery in highly complex situations, but can be an extra safety for the surgeon to double check for breaks and iatrogenic breaks as well.

Further studies with a larger number of patients and subgroup analysis of the above named baseline characteristics are needed to show whether the choice of scleral indentation method at the end of surgery indeed has an impact on the re-detachment rate or not.

## Supplementary Information

**Additional file 1: Video S1.** A detailed intraoperative view comparing both methods for scleral indentation in a more comprehensive way.

## Data Availability

The datasets generated during and/or analyzed during the current study are available from the corresponding author on reasonable request (Additional file 1).
